# The spatiotemporal localization of JAM-C following sciatic nerve crush in adult rats

**DOI:** 10.1002/brb3.63

**Published:** 2012-07

**Authors:** Parizad Avari, Wenlong Huang, Sharon Averill, Bartomeu Colom, Beat A Imhof, Sussan Nourshargh, John V Priestley

**Affiliations:** 1 Centre for Neuroscience and Trauma, Blizard Institute, Barts and the London School of Medicine and Dentistry, Queen Mary University of LondonLondon, United Kingdom; 2 Section of Anaesthetics, Pain Medicine, and Intensive Care, Department of Surgery and Cancer, Imperial College LondonSW10 9NH, United Kingdom; 3 William Harvey Research Institute, Barts and the London School of Medicine and Dentistry, Queen Mary University of LondonLondon, United Kingdom; 4 Medical Faculty, Centre Medical Universitaire, University of GenevaGeneva, Switzerland

**Keywords:** JAM-C, paranodes, peripheral nerve injury, remyelination, Schwann cells

## Abstract

JAM-C is a junctional adhesion molecule, enriched at tight junctions on endothelial and epithelial cells, and also localized to Schwann cells at junctions between adjoining myelin end loops. The role of JAM-C following peripheral nerve injury (PNI) is currently unknown. We examined the localization of JAM-C after sciatic nerve crush injury in adult rats. JAM-C immunoreactivity was present in paranodes and incisures in sham surgery control nerve, but distal to the crush injury significantly decreased at three and 14 days. JAM-C was re-expressed at 28 days and, by 56 days, was significantly increased in the distal nerve compared to controls. In a 7-mm length of sciatic nerve sampled distal to the crush site, the densities of JAM-C immunoreactive paranodes increased in the distal direction. Conversely, the densities of JAM-C immunoreactive incisures were highest immediately distal to the crush site and decreased in the more distal direction. Further analysis revealed a strong correlation between JAM-C localization and remyelination. Fifty-six days after crush injury, greater densities of JAM-C paranodes were seen compared to the nodal marker jacalin, suggesting that paranodal JAM-C precedes node formation. Our data are the first to demonstrate a potential role of JAM-C in remyelination after PNI.

## Introduction

JAM-C belongs to the immunoglobulin superfamily class of junctional adhesion molecules, composed of JAM-A, -B, -C, ESAM, CAR, and JAM4. It is characterized by two immunoglobulin folds in the extracellular domain (Ebnet et al. 2004), and is specifically enriched at tight junctions of cell–cell contacts. To date, JAM-C has largely been studied in the context of inflammatory and vascular events, due to its expression on endothelial cells and the cell surface of human platelets and certain leukocyte subtypes. Reflecting this wide expression pattern, JAM-C has been implicated in numerous events such as leukocyte trafficking, regulation of cell polarity, vascular permeability, angiogenesis, and cell–cell interactions (Aurrand-Lions et al. 2001; Gliki et al. 2004; Orlova et al. 2006; Bradfield et al. 2007; Weber et al. 2007; Woodfin et al. 2011). Recently, it has been shown that JAM-C is also expressed in peripheral nerves and that its expression is localized to Schwann cells at junctions between adjoining myelin end loops (Scheiermann et al. 2007; Colom et al. 2012) and to perineural cells (Colom et al. 2012). JAM-C has also been reported to be expressed in various tissues including intestine, skin, heart, lymph nodes, testis, thymus, lung, kidney, liver, placenta, and retina (Bazzoni 2003; Gliki et al. 2004; Aurrand-Lions et al. 2005; Ludwig et al. 2005; Daniele et al. 2007; Imhof et al. 2007; Scheiermann et al. 2009). JAM-C can engage in homotypic JAM-C/JAM-C or heterotypic JAM-C/JAM-B transinteractions (Arrate et al. 2001; Liang et al. 2002; Chavakis et al. 2004; Lamagna et al. 2005b). However, the interacting ligand in peripheral nerves is unknown.

JAM-C deficiency in peripheral nerves alters the integrity of the myelin sheath and consequently causes defective nerve conduction (Scheiermann et al. 2007), indicating that JAM-C plays an important role in myelination and/or myelin stabilization. Biopsies obtained from patients with chronic inflammatory demyelinating polyneuropathy displayed reduced numbers of JAM-C immunoreactive paranodal regions, in accord with a demyelinated state of nerve.

In the peripheral nervous system (PNS), myelinating Schwann cells wrap around axons, organizing the axonal membrane into distinct domains (Salzer 2008; Susuki and Rasband 2008). Efficient propagation at the nodes of Ranvier is facilitated by tight interactions between the axon and glial cells at the paranodal regions and between adjacent membrane layers of individual glial cells (autotypic junctions) (Poliak and Peles 2003). JAM-C expression in peripheral nerve lies selectively along the whole length of the paranodal terminal loops (Scheiermann et al. 2007), similar to other components of autotypic junctions, such as claudin-19 (Miyamoto et al. 2005). JAM-C is also enriched in Schmidt–Lantermann incisures, that is, conical tube-like structures that spiral through regions of compact lamellae. Since these noncompacted regions connect the Schwann cell body and the periaxonal cytoplasm, they are thought to act as highways for transport of metabolic substances (Berger and Gupta 2006).

Following crush injury, nerve fibers distal to the lesion degenerate in a process known as Wallerian degeneration. Schwann cells dedifferentiate, reversing molecular expression from a mature myelinating state to an immature one (Jessen and Mirsky 1999), and express surface molecules resulting in a permissive environment for axonal regeneration and remyelination (Abercrombie and Johnson 1946; Fawcett and Keynes 1990; Muller and Stoll 1998). As the Schwann cells begin to form myelin lamellae on the axon, the molecular phenotype switches back to its mature type. Schwann cells thus play an important role following nerve injury, not only in supporting axon regeneration but also in remyelinating regenerated axons. Since JAM-C is expressed by myelinating Schwann cells, and the JAM-C knockout has disrupted myelin (Scheiermann et al. 2007), we hypothesize that JAM-C may play an important role in the process of remyelination after nerve injury. To elucidate this hypothesis, we have examined the spatial and temporal patterns of JAM-C localization during peripheral nerve regeneration and remyelination of the rat sciatic nerve. To our knowledge, this is the first study to examine the effect of nerve injury on JAM-C localization.

## Methods and Materials

### Animals and surgical procedures

Adult male Wistar rats (225–250 g) were deeply anesthetized in a fume box using 5% isofluorane (Meril, Essex, UK), in addition to a mixture of oxygen and nitrous oxide (1:1 ratio), at a flow rate of 750–1000 mL/min. Subsequent anesthesia throughout the procedure was maintained using 2–2.5% isofluorane, with oxygen and nitrous oxide at an unchanged ratio, delivered through a nose-piece. The left sciatic nerve was exposed at mid-thigh level. A crush injury was induced to the sciatic nerve for 15 sec, using a pair of microforceps. After injury, the site of crush was marked with sterilized ink. Then the overlying muscle and skin were sutured, and the animals were returned to warm cages to recover from anesthesia. All experimental protocols were approved by the local ethics committee and in accordance with the U.K. Home Office regulations (Animals Act 1986).

### Perfusion and tissue processing

At three, 14, 28, or 56 days postsurgery (*n*= 4 per time point), animals were overdosed with sodium pentobarbital (60 mg/kg; Sagatal, France) and perfused via the ascending aorta with 4% paraformaldehyde in 0.1 M phosphate buffer, pH 7.4. A piece of the left sciatic nerve was dissected out, spanning from at least 5 mm proximal to the crush site to about 10 mm distal to the site, where the sciatic nerve starts to branch. The tissue was postfixed in the same fixative for 2 h at room temperature (RT), and then cryoprotected in 30% sucrose solution in 0.01 M phosphate buffered saline (PBS) overnight at 4°C. All sciatic nerves were then embedded in OCT medium (BDH Laboratory Supplies, Poole, UK) at –20°C and stored at –80°C until further processing. Four additional control rats, which had sham operations, were also processed in this way. Serial longitudinal sections of 8-μm thickness were cut using a cryostat, and consecutive sections processed for immunohistochemistry.

### Antibodies and immunohistochemistry

The following primary antibodies were used: mouse anti-200-kDa neurofilament (an axonal marker; N52 clone, Sigma, Gillingham, Dorset, UK; 1:1000), mouse anti-P0 (a peripheral myelin marker; clone 18 against aa32–38 of P0; Astexx, Austria; 1:3000), mouse anti-S100 (a Schwann cell marker; Sigma, UK; 1:2000), mouse antimyelin-associated glycoprotein (MAG, a marker for incisures; Chemicon, Temecula, CA; 1:200), mouse anti-pan Na_V_ channels (a nodal marker; Sigma, UK; 1:50), rabbit anti-JAM-C polyclonal anti-body (1:800∼1:1500). Lectin staining was performed using the fluorescein-labeled jackfruit agglutinin (jacalin, a nodal marker; Vector, Servion, Switzerland; 1:100). Appropriate secondary antibodies conjugated with Alexa Fluor dyes were purchased from Invitrogen (Grand Island, NY, USA) as follows: goat anti-rabbit Alexa Fluor 488 and goat anti-mouse Alexa Fluor 586 (all at 1:400). Hoeschst 33342 (Sigma, UK; 0.2 g/100 mL PBS) was used to reveal cell nuclei. All primary and secondary antibodies as well as the normal goat serum (NGS) were diluted in PBS containing 0.2% Triton X-100 and 0.1% sodium azide. The PBS wash (3 × 10 min) was a standard routine after incubation with a primary or secondary antibody. All incubation was carried out in a humidified chamber at RT.

The general procedure for immunohistochemistry was as follows. Following incubation with 10% NGS for 1 h, slides were incubated overnight with the anti-JAM-C antibody. Sections were then incubated with anti-rabbit secondary antibody for 2 h. After PBS washes, sections were then incubated with either anti-S100, anti-N52, anti-pan Na_V_, or anti-MAG antibody overnight. After 2 h incubation with anti-mouse secondary antibody, followed by PBS washes, sections were then counterstained with Hoeschst for 2 min. The slides were then rinsed with distilled water before final mounting in PBS glycerol (1:8). For P0 and JAM-C double labeling, sections were first treated with ice-cold methanol (–20°C) for 10 min. After incubation with the P0 primary antibody followed by Alexa Fluor 568, sections were incubated with the JAM-C primary antibody, followed by incubation with Alexa Fluor 488. For double labeling after lectin staining, NGS was applied for 1 h after 15-min jacalin application, and was then followed by primary and secondary antibodies using routine methods as described above. Specificity was confirmed in controls by incubating with secondary antibodies after omission of the primary antibodies. The characteristics of the anti-JAM-C antibody have been reported previously (Lamagna et al. 2005a) and its specificity has also been tested using JAM-C KO mice (Scheiermann et al. 2007, 2009).

Sections were viewed on a Leica epifluorescence microscope (Wetzlar, Germany) using appropriate filter blocks (TRITC, FITC, or DAPI). Images were taken using a Hamamatsu C4742–95 digital camera (Herrsching, Germany) and the Leica QWin program (Leica, Germany). Figures were prepared using Adobe Photoshop CS2.

### Morphometric and quantitative analysis

In the injured rat sciatic nerve, the crush site was relatively easy to identify based on the marked reductions of P0, N52, or JAM-C staining. P0 images were taken at ×20 objective magnification at three areas: namely 1.4-, 4.0-, and 6.6 mm distal to the crush site. Images were also taken 1.4 mm proximal to the crush site.

The Leica QWin software was used to quantify the P0 immunostained myelin by converting the camera image into a binary image of the P0 labeling. Three measuring frames of identical size (640 μm × 640 μm) were then randomly applied onto each image, and the percentage of the measuring frames covered by this binary image was determined. The mean of these three measures was then determined for each area per animal. Regions were analyzed from at least three sections per animal. In some distal nerve areas, myelin debris was manually excluded in Photoshop and then the above quantification performed. This method of analysis was chosen because P0 labeling was too extensive to allow for unambiguous identification of individual axons and myelin. However the analysis does not distinguish between decreased P0 labeling due to thin myelin, and decreased P0 labeling due to decreased space occupied by myelinated nerve fibers.

For analysis of JAM-C localization, images were taken at ×40 objective magnification at each location as described above. In Photoshop, a counting grid was placed over each image and the total number of JAM-C immunoreactive paranodes or incisures was counted. Jacalin immunoreactive or Na_V_ immunoreactive nodes and MAG immunoreactive incisures were counted in a similar way as for JAM-C. The results were expressed as densities (e.g., the number of JAM-C immunoreactive paranodes/mm^2^). Regions were analyzed from at least six sections per animal.

### Statistical analysis

All results are expressed as mean ± standard errors of the means (SEM). One-way, two-way, or repeated measures of analysis of variance (ANOVA) were used when appropriate. The Tukey–Kramer multicomparison adjustment was used as the post-hoc test to calculate the significance levels. *P* < 0.05 was considered statistically significant.

## Results

### JAM-C localization in normal sciatic nerve

**Figure 1:**
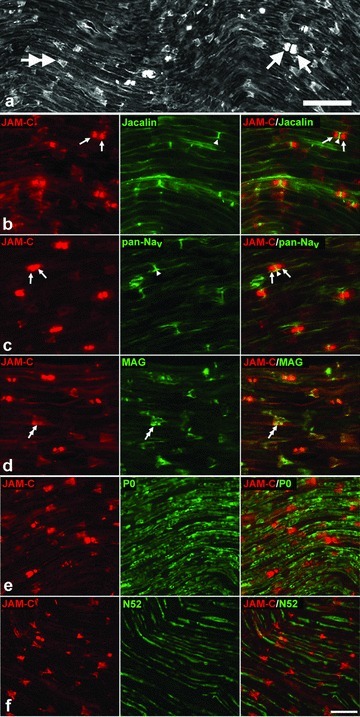
JAM-C localization in sham surgery control rat sciatic nerve. (a) JAM-C immunostaining in the peripheral nerve with labeling of paranodes (arrows) and incisures (double-arrows). Rows (b–f) show a sequence of double labeling with JAM-C to illustrate its relationship with other structures of the nerve. (b, c) JAM-C paranodes (arrows) are restricted to areas surrounding the nodes of Ranvier (arrowheads), as indicated by immunostaining with nodal markers, jacalin and pan-Na_V_. (d) colocalization of JAM-C immunoreactive incisures with MAG immunoreactive incisures (double arrows), confirming JAM-C localization at the incisures. (e) JAM-C double staining with P0 (a marker for compact myelin), confirming JAM-C localization of paranodes and incisures to regions that lack compact myelin. (f) double labeling with N52 neurofilament antibody, revealing the spatial relationship between JAM-C immunoreactive paranodes and incisures and neurofilament immunoreactive axons. Scale bars = 50 μm (a), 25 μm (b–p).

Immunohistochemistry on longitudinal sections of sciatic nerves of sham surgery control adult rats demonstrated JAM-C localization in peripheral nerves (Fig. 1a). Double labeling with two markers of nodes of Ranvier (jacalin and pan-Na_V_) and with a marker of Schmidt–Lantermann incisures (MAG) confirmed that JAM-C is concentrated in paranodal regions of nerves (Fig. 1b and c) and in Schmidt–Lantermann incisures (Fig. 1d). Double labeling with antibodies to neurofilament and to P0 confirmed that JAM-C immunoreactive structures are associated with axons and with regions that lack compact myelin (Fig. 1e and f).

### Sciatic nerve crush induces changes in JAM-C localization

In order to examine the localization of JAM-C after peripheral nerve injury (PNI), immunolabeling followed by quantitative analysis of paranodes and incisures was performed spatially in the near, mid-, and far-most distal regions (1.4, 4.0, and 6.6 mm, respectively, from the crush site) along the distal nerve. Additionally, this localization was examined temporally at various time points; namely three, 14, 28, and 56 days after nerve crush. These time points were selected as covering both the degeneration stage (three days) and the remyelination process, which is known to begin within two weeks of the onset of axonal regeneration in rats (Burnett and Zager 2004).

### The spatiotemporal localization of JAM-C immunoreactive paranodes in the regenerating nerve

**Figure 2:**
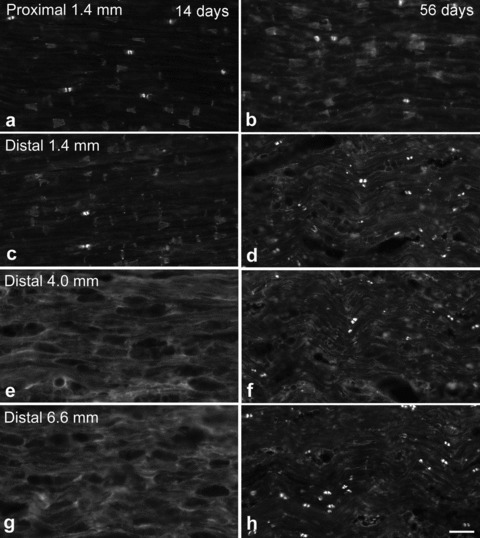
JAM-C spatial localization at 14 and 56 days after crush injury. 1.4 mm proximal to the crush site (a), JAM-C localization at 14 days is similar to that observed in the controls. 1.4 mm distal to the crush (c), there is significantly lower JAM-C immunoreactivity, while in the mid- and far-most distal regions (e, 4 mm; g, 6.6 mm), there is complete disappearance of JAM-C localization, with no paranodes or incisures visible. By 56 days after injury, JAM-C localization has recovered in all three levels of the distal nerve (d, f, h), and particularly at the 6.6 mm distal level (h) where the number of JAM-C immunoreactive paranodes appears even greater than proximal to the crush site (b). However the size of the paranodes and incisures in the distal nerve appears smaller than those observed in the region proximal to the crush. Scale bar = 50 μm.

**Figure 3:**
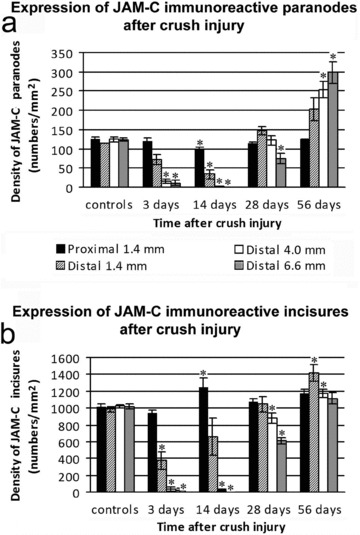
Quantification of JAM-C localization in sciatic nerve in the controls and after crush injury. The density of JAM-C immunoreactive paranodes (a) and incisures (b) proximal and distal to a sciatic nerve crush site is shown for the controls and at four different time points postinjury. Values represent means ± SEM. *Significant difference (*P* < 0.05) versus the controls.

At three (not illustrated) and 14 days (Fig. 2a, c, e, and g) after injury, JAM-C immunoreactive paranodes appeared to be decreased distal to the crush site, and this decrease was confirmed by quantitative analysis (Fig. 3a). In the distal region closest to the crush site (1.4 mm distal), the density of JAM-C immunoreactive paranodes was decreased at three days, but this decrease was not statistically significant. However, by 14 days there was a significant reduction in JAM-C immunoreactivity (Fig. 2c), which corresponded to a 70% decrease in paranodal JAM-C when compared to the controls (34 ± 11/mm^2^ vs. 115 ± 4/mm^2^; Fig. 3a; *P*= 0.004). The intermediate and far-distal regions (4.0 and 6.6 mm) showed an almost complete deterioration of JAM-C immunoreactive paranodes at both three and 14 days (Figs. 2e, g, and 3a). At 14 days following injury, there was also a small but significant decrease in the density of JAM-C immunoreactive paranodes in the nerve just proximal to the crush site (Figs. 2a and 3a). A significant spatial pattern of JAM-C localization was noted within the nerve at three and 14 days, with a progressive downregulation of JAM-C immunoreactive paranodes, which appeared first in the most distal region of the sampled nerve (6.6 mm) and spread retrogradely to the region closest (1.4 mm) to the crush site.

Twenty-eight days following injury, there appeared to be indications of recovery in the densities of JAM-C immunoreactive paranodes throughout the distal nerve (not illustrated). However, paranodes appeared narrower and shorter in size compared to uninjured nerves, and this was confirmed by quantitative analysis (Table 1). JAM-C immunoreactive paranode numbers were similar in the near-distal region to those in the controls, albeit paranodal density in the far-most distal region was 40% lower than the controls (Fig. 3a; *P* < 0.05). In contrast to the loss of JAM-C immunoreactivity following earlier time points, we observed a substantial increase of JAM-C paranodal immunoreactivity at 56 days in the distal nerve as compared with either the controls or the proximal region of the nerve (Fig. 2b, d, f, and h). The paranodes remained small in size (Table 1), similar to those observed at 28 days after injury (Fig. 2d, f, and h). At 1.4 mm distal to the crush site, in comparison to the controls, there was a 77% increase in paranodal density, but this was not statistically significant (Fig. 3a). Meanwhile, in the more distal regions at 4.0 and 6.6 mm, the numbers had increased significantly by 104% and 142%, respectively, in comparison to the controls (253 ± 22 paranodes/mm^2^ vs. 124 ± 7 paranodes/mm^2^ for the 4.0-mm region; 298 ± 28 paranodes/mm^2^ vs. 123 ± 4 paranodes/mm^2^ for the 6.6-mm region; Fig. 3a). Bordering significance was observed comparing the near- and far-most distal regions, emphasizing the spatial pattern of JAM-C localization along the injured nerve (*P*= 0.05; Fig. 3a).

**Table 1:** Paranode and incisure measurements

	Paranode		Incisure	
		
	Length	Width	Length	Width
Sham surgery controls	7.38 ± 0.87	4.21 ± 0.20	9.33 ± 0.16	6.99 ± 0.34
14 days after crush injury	3.56 ± 0.74*	3.57 ± 0.62	7.41 ± 0.30*	4.91 ± 0.23**
28 days after crush injury	5.30 ± 0.35	2.76 ± 0.06	5.74 ± 0.47***	4.01 ± 0.33**
56 days after crush injury	5.76 ± 0.16	3.31 ± 0.24	5.54 ± 0.37***	4.16 ± 0.27**

Paranode and incisure measurements in controls and after crush injury, 1.4 mm distal to the injury site. Each value is the mean ± SEM of three animals. The animals with sciatic nerve crush have significantly reduced sizes of paranodes and incisures when compared to those of the noncrush control animals (*P < 0.05, **P < 0.01, and ***P < 0.001, all compared to sham control).

### The spatiotemporal localization of JAM-C immunoreactive incisures

JAM-C immunoreactive incisures were examined in a similar spatiotemporal manner as JAM-C immunoreactive paranodes. At three (not illustrated) and 14 days (Fig. 2a, c, e, and g) after injury, JAM-C immunoreactive incisures decreased significantly in the distal nerve and remained below control levels (Fig. 3b). Fourteen days postcrush, incisural shapes had become much narrower (Table 1), and the interincisural distance appeared to have decreased (Fig. 2c). Similar to our findings with paranodes, the complete disappearance of JAM-C immunoreactive incisures was apparent in the middle and far-most distal regions at three and 14 days (Figs. 2e, g, 3b). Analogous to the paranodes, a spatial pattern of localization after injury along the length of the distal nerve was observed for the incisures, with the greatest loss of JAM-C appearing in the more distal regions. However, in contrast to the paranodes, at 14 days there was a significant increase in the number of incisures in the proximal nerve in comparison to controls (1245 ± 105 vs. 1012 ± 34 incisures/mm^2^; *P* < 0.05; Fig. 3b).

JAM-C immunoreactive incisures appeared to show numerical recovery by 28 days (Fig. 3b) after injury, similar to the findings of JAM-C localization in paranodes. The shapes of incisures remained narrow, but their length had also decreased (Table 1). This may correspond to “partial” Schmidt–Lantermann incisures (i.e., incisures that do not cross through the entire thickness of the sheath). In the nerve just distal (1.4 mm) to the crush site, the density of JAM-C immunoreactive incisures was similar to controls (1047 ± 93 incisures/mm^2^ vs. 986 ± 30 incisures/mm^2^; Fig. 3b). However, in the mid- and far-distal regions, there was still a significant decrease (16% and 40%, respectively, compared to the controls; *P* < 0.05).

Fifty-six days following nerve injury (Fig. 2d, f, and h), the incisures remained small (Table 1), similar to those observed at 28 days. Quantitative analysis showed the density of JAM-C immunoreactive incisures was higher at just-distal site (1.4 mm) compared to controls, but decreased along the length of the nerve from the crush site to reach normal levels in the far-distal regions, with 1417 ± 93 JAM-C immunoreactive incisures/mm^2^ in the near-distal region compared to 1114 ± 65 JAM-C immunoreactive incisures/mm^2^ in the far-distal region (*P* < 0.05; Fig. 3b). This pattern of localization spatially is the opposite to that observed with the JAM-C immunoreactive paranodes.

Summarizing all results of crush lesions at various time points, the densities of JAM-C immunoreactive paranodes and incisures decreased three days following nerve crush injury, and then notably underwent a subsequent increase over time. Through the majority of the injured nerves, a significant spatial pattern of JAM-C localization for both paranodes and incisures existed, emphasizing the sequential course of degeneration and regeneration.

### JAM-C localization correlates with remyelination after crush injury

**Figure 4:**
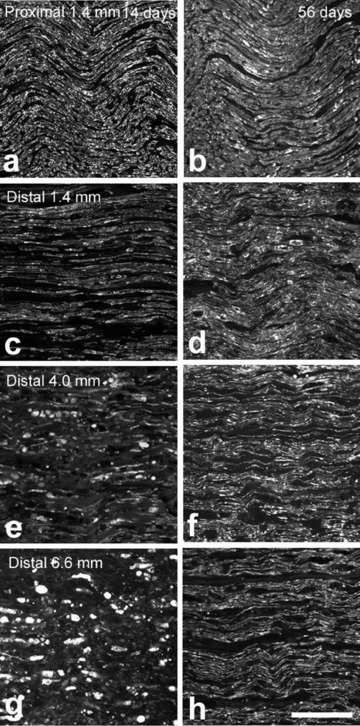
Remyelination along peripheral nerves following crush injury. Micrographs showing P0 immunofluorescence at various lengths along a nerve at 14 days (a, c, e, g) and 56 days (b, d, f, h) after crush injury. The images illustrate the progressive nature of remyelination proceeding in an anterograde direction along the injured nerve, at each time point. The degree of myelination is greater nearer the crush site (compare c with g, or d with h) and increases with time (compare d with c, or h with g). Note also, myelin debris is more prominent in the far-distal nerve and at the earlier time point (e, g). Scale bar = 100 μm.

In order to examine the relationship between JAM-C localization and remyelination after PNI, we performed a detailed analysis of the time course of myelin localization. Immuno-labeling with anti-P0 antibody, a marker of peripheral myelin, was performed at various time points after nerve crush. In longitudinal sections, axons proximal to the crush site were revealed to have continuous and regular layers of myelin (Fig. 4a and b), similar to that observed in intact control nerve (Fig. 1e).

**Figure 5:**
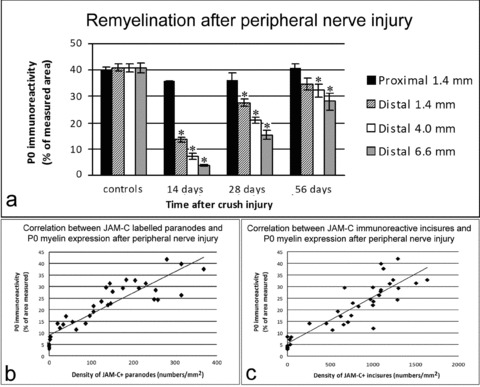
Localization of JAM-C immunoreactive paranodes and incisures correlates with the remyelination process. The histogram (a) shows the spatiotemporal localization of myelin after crush injury. The densities of P0 immunoreactivity are expressed as the percentage (%) of measured area. Values represent means ± SEM. *Significant difference (*P* < 0.05) versus controls. The graphs showing the densities of JAM-C immunoreactive paranodes (b) and incisures (c) in relation to P0 myelin localization reveal a pronounced relationship. Spearmann's rank tests confirmed the positive correlation between P0 immunoreactivity and both JAM-C immunoreactive paranodes and incisures (*r*= 0.897 and *r*= 0.885, respectively; *P* < 0.05). Data from all time points and all distal segments after injury have been included, but not from the proximal segments.

A reduced level of P0 staining was observed at 14 days following injury, with the continuous myelin layers having disappeared distal to the crush site (Fig. 4c, e, and g). A dis-orderly pattern of P0 localization was present, with visibly large amounts of myelin debris particularly in the far-most distal region (Fig. 4g). Quantitative analysis revealed a progressive reduction of P0 immunoreactivity along the length of the nerve distal to the crush site (Fig. 5a). In the near-distal area, there was a 67% reduction in P0 immunoreactivity compared to the controls (P0 density: 13.6 ± 0.8% vs. 40.9 ± 1.3%; *P* < 0.05), whereas in the far-distal region there was a 91% reduction in P0 localization (P0 density: 3.7 ± 0.8% vs. 40.8 ± 1.3%; *P* < 0.05). This spatial pattern of localization closely resembles that observed with JAM-C immunostaining.

With the progressive nature of the remyelination process, in comparison to 14 days, 28 days after injury showed a greater degree of remyelination in the distal nerve (not illustrated). However, there yet remained a 33% decrease in the near-distal nerve, with a 62% decrease in the far-distal nerve (Fig. 5a; *P* < 0.05). By 56 days (Fig. 4b, d, f, and h), further remyelination had occurred across the injured nerve, with levels of myelin in the near-distal regions comparable to that in the intact nerve controls (Fig. 5a). However, in the far-most distal region, the level of remyelination had not yet reached that of the controls, that is, myelin density remained reduced by 31% (P0 density = 28.2% compared to 40.8% in the controls; Fig. 5a).

At each time point, the density of both JAM-C immuno-reactive paranodes and incisures appeared to follow the course of myelination. A Spearmann's rank test (Fig. 5b and c) confirmed that after injury there is a close correlation between levels of P0 immunoreactivity and the density of JAM-C immunoreactive paranodes and incisures (*r*= 0.897 and *r*= 0.885 for paranodes and incisures, respectively).

### Increased proportion of JAM-C immunoreactive paranodes at 56 days

**Figure 6:**
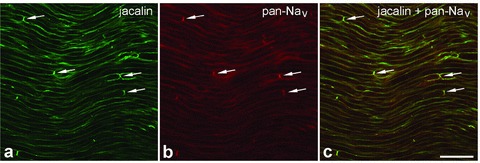
Jacalin as a nodal marker. Double labeling with the lectin jacalin (a) and a pan-Na_V_ antibody (b) gives extensive double labeling of nodes of Ranvier (arrows). No difference was seen in the nodal localization of the two markers. Scale bar = 50 μm.

In order to determine the proportion of total paranodes and incisures that are JAM-C immunoreactive, JAM-C was double immunostained with jacalin, a marker of nodes (K. Smith, pers. comm.), and with MAG, a marker for incisures. In separate experiments, jacalin was shown to be expressed at nodes of Ranvier, as observed through double labeling with the well-established marker, pan-Na_V_ (Fig. 6). Moreover, the jacalin staining process was short and reliable. Hence, we used jacalin as the nodal marker in the current study.

**Figure 7:**
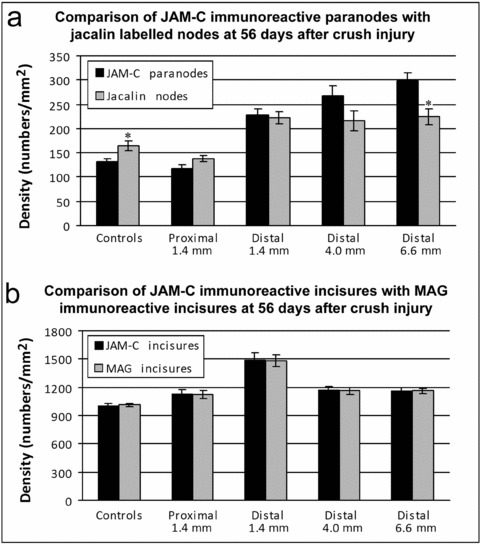
Proportions of JAM-C immunoreactive paranodes and incisures in the controls and 56-day crush injury sciatic nerve. The histograms demonstrate (a) the density of JAM-C immunoreactive paranodes compared with jacalin-labeled nodes, and (b) the density of JAM-C immunoreactive incisures in relation to the density of MAG immunoreactive incisures. In the controls, approximately 99% of incisures express JAM-C, whereas only 77% of paranodes express JAM-C. At 56 days, in the regenerating nerve distal to the crush, the ratio of JAM-C immunoreactive incisures remains unchanged, however there are greater numbers of JAM-C immunoreactive paranodes than jacalin-labeled nodes. Values represent means ± SEM. *Significant difference (*P* < 0.05) versus controls.

**Figure 8:**
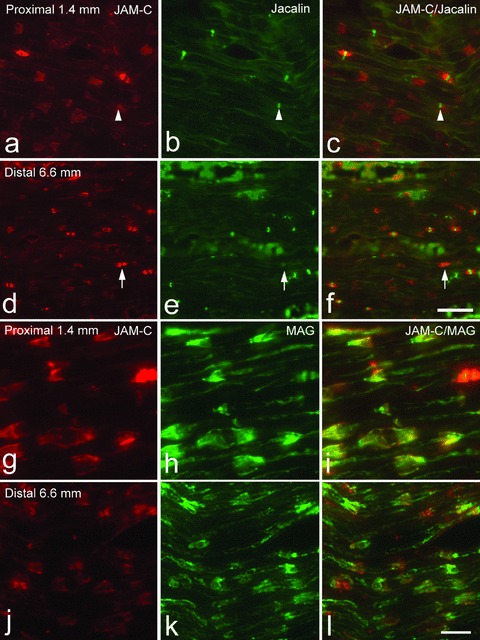
JAM-C localization in the proximal and distal nerve at 56 days after injury in comparison to jacalin-labeled nodes (a–f) and MAG immunoreactive incisures (g–l). In the proximal nerve (a–c), there are more jacalin nodes, in comparison to JAM-C immunoreactive paranodes (arrowhead indicates a jacalin node without a JAM-C immunoreactive paranode). However in the far-distal region (d, f), 6.6 mm from the crush, there appear to be greater densities of JAM-C immunoreactive paranodes in comparison to jacalin-labeled nodes (arrow indicates a JAM-C immunoreactive paranode without a jacalin node). In contrast, in both proximal (g–i) and distal (j–l) regions of the nerve, there is good colocalization between JAM-C and MAG immunoreactive incisures. Scale bars = 100 μm (a–f), 50 μm (g–l).

Our results in controls showed that not all of the paranodal regions were positive for JAM-C, with further quantification showing only ∼80% of the total jacalin immunoreactive nodal regions were surrounded by JAM-C immunoreactive paranodes (132 ± 7 JAM-C immunoreactive paranodes vs. 165 ± 11 jacalin immunoreactive nodes; *P* < 0.05; Fig. 7a). In contrast, double labeling for JAM-C and jacalin 56 days after injury (Fig. 8a–f) revealed a significantly disproportionate increase in the number of JAM-C immunoreactive paranodes in the far-most distal region compared to jacalin immunoreactive nodes (Fig. 7a). Intriguingly, the proportion of JAM-C immunoreactive paranodes without jacalin nodes increased progressively through the distal region (% JAM-C immunoreactive paranodes without jacalin nodes in far-distal region =∼25%; *P* < 0.05). Throughout the injured nerve, we hardly ever observed jacalin nodes without JAM-C paranodes.

We also compared the proportion of JAM-C immuno-reactive incisures with MAG immunoreactive incisures. In controls, no difference was observed between the two groups, and the ratios remained unchanged even after injury (Figs. 7b and 8g–l).

## Discussion

In the current study, we have investigated the localization of JAM-C in the PNS using an injury model that comprises a sciatic nerve crush. We have shown that JAM-C is expressed in myelinating Schwann cells and is localized to the paranodes and incisures in adult rats, consistent with the reports by Scheiermann and Colom (Scheiermann et al. 2007; Colom et al. 2012). We have also demonstrated the spatiotemporal localization of JAM-C following sciatic nerve crush injury in rats, and shown a strong correlation with the process of remyelination after injury. Furthermore, we have shown that, at 56 days after injury, there is a persistent upregulation of JAM-C in the distal nerve and the morphology of JAM-C immunoreactive paranodes and incisures appeared abnormal compared to the controls. In addition, we observed significant numbers of JAM-C immunoreactive paranodes that did not express the nodal marker, jacalin, and this may indicate that JAM-C localization precedes node formation.

### The spatiotemporal pattern of JAM-C localization after sciatic nerve injury

Our observations at various time points postinjury demonstrate a transient loss of JAM-C localization followed by a progressive gradual reappearance, which is in keeping with previous studies of paranodes (Burnett and Zager 2004). This temporal pattern of localization was further corroborated by the changes expressed spatially ranging from the highest levels of JAM-C close to the site of crush, with ensuing decline through the remaining distal nerve. This occurred at all time points except the longest (56 days), when the density of JAM-C immunoreactive paranodes was highest in the most distal region of the nerve.

Three days after injury, we expected that the level of JAM-C localization would have decreased dramatically in the distal nerve. However, although JAM-C localization was very low in the most distal regions, there remained significant JAM-C localization in the region just distal to the crush site. It is probable that this pattern of JAM-C localization was caused by Wallerian degeneration progressing in a retrograde direction with fragmentation beginning at the distal end, resulting in slightly higher numbers of JAM-C immunoreactive paranodes and incisures at the near-distal region than more distally along the nerve. The progressive nature of Wallerian degeneration has long been controversial, with conflicting reports over the directionality and whether the distal stumps of the injured axons degenerate anterogradely, retrogradely, or simultaneously (reviewed in Joseph 1973; Lubinska 1975; Chaudhry et al. 1992). Consistent with recent reports (Lunn et al. 1990; Beirowski et al. 2005), we show the progressive nature of Wallerian degeneration begins distally after crush injury. The reason for this is not known, but may be due to the distal end of the nerve being more vulnerable to compromised anterograde axonal transport (Beirowski et al. 2005).

At later time points (e.g., 28 days), the proximo-distal gradient of JAM-C localization is consistent with regeneration proceeding in the anterograde direction, and Schwann cells re-expressing JAM-C as they mature into a myelinating phenotype. It is well known that Schwann cells classically undergo phenotypic modulation during development and in response to injury (Fawcett and Keynes 1990; Kioussi et al. 1995). During development, JAM-C localization is absent in immature Schwann cells and is only expressed from postnatal day P5 onwards, as observed in mice by Scheiermann et al. (2007). Our study adds another example to the literature of developmental recapitulation postinjury, demonstrated by the downregulation and subsequent upregulation of JAM-C.

### Chronic JAM-C localization of paranodes and incisures after injury

At 56 days postinjury, significantly increased numbers of JAM-C immunoreactive paranodes were present in the region distal to the crush site, with JAM-C immunoreactive paranodal densities highest in the far-most distal region; almost 2.5-fold compared to numbers in uninjured sciatic nerve. This trend may be explained by abnormally short internodal distances, thus resulting in increased numbers of JAM-C immunoreactive paranodes. Abnormally short internodal distances have been implicated in causing a conduction velocity lag in regenerated axons, as they are formed by more than a threefold increase during Schwann cell proliferation in the distal nerve stump (Hiscoe 1947; Haftek and Thomas 1968). These distances slowly increase during the course of myelin sheath remodeling by Schwann cells (Hildebrand et al. 1994; Schafer et al. 2006). Hence, regions in the far-most distal regions may have more paranodes, as a result of varying degrees of myelin sheath remodeling through the distal nerve. Previous studies indirectly support these findings by observations of increased nodes following crush injury (Nakata et al. 2008). It would be interesting to look at survival times longer than 56 days, to determine how long it takes JAM-C localization to return to naïve levels.

In contrast, the JAM-C immunoreactive incisural densities decreased proximo-distally, with the highest numbers in the near-distal region. As incisures subserve a role to maintain myelin sheath integrity, it is likely that the increased numbers are present to help provide stability between the various myelin sheath layers. Their smaller size, postinjury, may be related to thin myelin during remyelination. The localization of JAM-C is specific to noncompact myelin; that is, at the incisures and paranodes, where a wide variety of specialized junctions exists, including gap, adherens, and tight junctions. These regions are believed to be critical for signaling, transport of small metabolites, and maintenance of myelin structure (Spiegel et al. 2007). JAM-C may play such a role in promoting the maintenance of myelin structural integrity.

From our measurements of P0 myelin density, at the most distal region at 56 days, myelination had not yet reached the levels of controls. This implies that the increased JAM-C localization may possibly be due to the remyelinating nerve still being present in a remodeling stage. In rats, 56 days remains a relatively long period of time for recovery after injury, and it is potentially possible that a chronic pathological state may have been reached, in which there is a disproportionate ratio of paranodes to incisures. Consistent with our results, walking track analysis has shown that even after eight weeks, recovery after sciatic nerve crush in rats is only 40% of that of controls (Vogelaar et al. 2004).

### Role of JAM-C paranodes in PNS node of Ranvier formation after injury

Another interesting finding of this study is that in uninjured nerves, JAM-C paranodal localization did not surround all of the nodal regions. It is perhaps for this reason that in JAM-C knock-out mice, only a proportion of fibers exhibit layers of loose myelin within the periaxonal space forming tomacula (Scheiermann et al. 2007). Alternatively, jacalin may label some unmyelinated fibers that are known not to express JAM-C (Colom et al. 2012). However, 56 days after injury, not all of the JAM-C paranodal regions were positive for jacalin nodal staining, highlighting the difference between mature nerves and regenerating nerves. It is possible that the mismatch between JAM-C and jacalin is due to jacalin not staining immature nodes. This possibility should be examined by double labeling with Na_V_ at different survival times post-injury. However, another interesting possibility is that paranodal JAM-C is expressed ahead of the nodal structure during the development of the paranodal–nodal region. Indeed, in the CNS, paranodal proteins, including neurofascin, appear to cluster before node formation and facilitate ion-channel clustering (Rasband et al. 1999; Schafer et al. 2004). In the PNS, studies using the paranodal marker, Caspr, have argued in favor of a model in which paranodal contact follows node formation (Susuki and Rasband 2008). However sometimes paranodal neurofascin is detected before Na_V_ clustering, indicating the presence of multiple mechanisms contributing to node formation in the PNS (Schafer et al. 2006). To further probe the role of JAM-C, it would be interesting to carry out double labeling with markers specific for autotypic junctions and for axon–glial junctions at the paranode. Despite the findings of this study, it is unlikely that JAM-C itself is a key regulator for the formation of nodes after injury, as JAM-C global knockout-mice showed that the clustering of Na_V_ channels was not affected by JAM-C deficiency (Scheiermann et al. 2007) and Schwann cell specific JAM-C knockouts show only modest increases in nodal length (Colom et al. 2012).

### JAM-C localization parallels remyelination

During the regenerative period (14, 28, 56 days), we showed that JAM-C localization correlated highly significantly with the degree of P0 myelin localization, with greater JAM-C localization associated with increased remyelination. We have also shown, as discussed above, that JAM-C localization in paranodes may precede node formation. Our results indicate that JAM-C may play an important role in node formation and/or myelin localization, and it would be interesting to search for ligands that interact with JAM-C, to determine the mechanisms that underlie Schwann cell to Schwann cell JAM-C signaling. However, further studies are required to establish a direct causal relationship. Colom et al. (2012) have recently reported the generation of a transgenic mouse with JAM-C selectively deleted from Schwann cells. Regeneration studies in the transgenic line would be very interesting and allow the effect of JAM-C deletion on myelination and node formation to be directly examined. Without such studies, our conclusions about the role of JAM-C in regeneration must remain tentative.
